# Diversity of *Blastocystis* subtypes in dogs in different geographical settings

**DOI:** 10.1186/1756-3305-6-215

**Published:** 2013-07-24

**Authors:** Wenqi Wang, Leigh Cuttell, Helle Bielefeldt-Ohmann, Tawin Inpankaew, Helen Owen, Rebecca J Traub

**Affiliations:** 1 School of Veterinary Science, The University of Queensland Gatton Campus, Queensland 4343, Australia; 2 Australian Infectious Disease Research Centre, The University of Queensland, Queensland, Australia; 3 Department of Veterinary Disease Biology, Faculty of Health and Medical Science, University of Copenhagen, Copenhagen, Denmark; 4 Department of Parasitology, Faculty of Veterinary Medicine, Kasetsart University, Bangkok, Thailand

**Keywords:** *Blastocystis*, Dog, Zoonosis, Epidemiology

## Abstract

**Background:**

*Blastocystis* is a ubiquitous, globally distributed intestinal protist infecting humans and a wide range of animals. Several studies have shown that *Blastocystis* is a potentially zoonotic parasite. A 1996 study reported a 70% *Blastocystis* prevalence in Brisbane pound dogs while another study found that pet dogs/cats of 11 symptomatic *Blastocystis* infected patients harboured at least one *Blastocystis* subtype (ST) in common with the patient. These results raised the possibility that dogs might be natural hosts of *Blastocystis*. In this study, we aimed to investigate this hypothesis by estimating the prevalence of *Blastocystis* carriage and characterising the diversity of STs in dogs from three different environmental settings and comparing these STs with the range that humans harbour.

**Methods:**

Two hundred and forty faecal samples from dogs from three different geographical regions with varying levels of socio-economic development and sanitation, namely i) 80 pet and pound dogs from Brisbane, Australia, ii) 80 semi-domesticated dogs from Dong Village, Cambodia and iii) 80 stray dogs from the densely populated cities of Sikkim, Delhi and Mumbai in India, were screened for *Blastocystis* using PCR and subtyped based on the “barcode region” of the small subunit ribosomal RNA (SSU rRNA) gene.

**Results:**

The prevalence of *Blastocystis* in dogs from Brisbane and Cambodia was 2.5% (2/80) and 1.3% (1/80), respectively, in contrast to 24% (19/80) in stray dogs from India. Stray dogs in India carried a diverse range of *Blastocystis* STs including ST 1, 4, 5 and 6 while the dogs from Brisbane carried only ST1 and one Cambodian dog carried ST2.

**Conclusion:**

The results suggest there is geographical variation in *Blastocystis* prevalence and STs between dog populations as reported in human studies. In addition, the greater diversity of STs and higher prevalence of *Blastocystis* in Indian stray dogs compared to pet/pound and community dogs in Australia and Cambodia could reflect close proximity to humans and other animals and exposure to their faeces. It appears that dogs are not natural hosts for *Blastocystis* but rather are transiently and opportunistically infected with a diversity of STs.

## Background

*Blastocystis* is a ubiquitous, intestinal protist with a high prevalence worldwide in humans and animals. Host types shown to carry *Blastocystis* include humans, non-human primates, a range of domesticated and wild mammals and birds [1]. *Blastocystis* is the most common gastrointestinal parasite recovered in human fecal parasite surveys, with prevalence ranging from 0.5% in developed countries to 60% in developing countries [2]. The most accepted proposed mode of transmission is the fecal-oral route either by direct contact or food and water-borne transmission [3-5]. Molecular analysis of the SSU-rRNA gene has allowed for subdivision of *Blastocystis* into 14 distinct subtypes (STs) in humans, non-human primates (NHPs), mammals and birds [1,6,7]. Humans have been shown to carry STs 1–9, with ST3 being the most prevalent followed by ST1 [8].

*Blastocystis* is a potential zoonosis as suggested by a number of studies that have isolated identical STs of *Blastocystis* from humans and their in-contact animals [3-6,9]. Recently, domestic dogs were proposed as a potential source of *Blastocystis* infection to humans [10]. Eleven symptomatic patients, their pets, as well as 59% of family members tested positive for *Blastocystis* by PCR and all infected family members and domestic animals (dogs and cats) harboured at least one *Blastocystis* ST in common with the patient. A study by Duda *et al*. [11] on *Blastocystis* prevalence in 72 domestic dogs (70 pound dogs and 2 pet dogs) in Brisbane showed a 70.8% prevalence using light microscopy on fecal wet mounts. These results raised the possibility that dogs might be natural hosts of *Blastocystis* and potential sources of zoonotic transmission to humans. In this study, we aimed to investigate this hypothesis by estimating the prevalence of *Blastocystis* infection and characterising the diversity of STs in dogs from three different environmental settings and comparing these STs with the known range harboured by humans.

## Methods

### Sampling

A total of 240 dogs were screened for *Blastocystis*, including 80 dogs from each of the 3 following settings 1) pound and pet dogs in Brisbane, a major metropolitan area in Queensland, Australia 2) semi-domesticated dogs from 36 households in Dong Village, Cambodia, 3) stray street dwelling dogs from the Indian cities of Delhi, Sikkim and Mumbai. The three settings differed from each other in terms of geographical location, level of hygiene and opportunities for dogs to come into contact with faeces from humans and other animals.

In Brisbane, freshly voided faecal samples were collected off the ground from pet and pound dogs. These samples were obtained in 2010 – 2011 from 45 pound dogs and 35 pet dogs. Samples were stored at room temperature until DNA extraction was performed, usually within 12 hours. In Cambodia, faecal samples were collected per-rectum from 80 semi-domesticated dogs from 36 households in the Dong village and preserved in 2.5% potassium dichromate (K_2_Cr_2_O_7_) (Sigma-Aldrich, Australia) until DNA extraction was performed. In India, stray dogs were sampled from three cities, namely Sikkim (n = 25), Delhi (n = 27) and Mumbai (n = 28) in 2008 as part of a street dog sterilization project run by Non-Governmental Organisations and municipalities. Faecal samples were collected from stray dogs per-rectum and preserved in 70% ethanol. This project was approved by the University of Queensland Animal Ethics Committee with approval no. ANRFA/472/11.

### Molecular analysis

#### DNA extraction

DNA was extracted from faeces using the QIAamp DNA Stool Mini Kit (Qiagen, Germany) with minor modifications. Following addition of buffer ASL and homogenisation, samples were subjected to freeze/thaw 3 times repeatedly in liquid nitrogen and 95°C water, followed by a further 5 mins incubation at 95°C to lyse cells.

#### Control testing of extracted DNA

For internal process control, all samples were tested using published universal primers that amplify a 140 bp fragment of the 18S ribosomal RNA gene from eukaryotic DNA to detect for amplifiable DNA [12]. The primers used were forward: 18SEUDIR 5′-TCTGCCCTATCAACTTTCGATGG-3′ and reverse: 18SEUINV 5′-TAATTTGCGCGCCTGCTG-3′. PCR cycling conditions were optimised by modifying the published real-time PCR protocol using an annealing temperature of 60°C. This testing was performed to check for inhibition and also ensure that the variation in the sample preservation methods would not affect the accuracy of results.

#### PCR amplification

Two previously published PCR primer sets and conditions were utilised for the detection and characterisation of *Blastocystis* STs [13-15]. A single step [14] and nested PCR [13,15] were performed on each sample using a Bio-Rad C1000 Thermal Cycler (Bio-Rad Laboratories, Inc., Hercules, USA) (Table 1) to amplify a 600 bp and 1100 bp region of the SSU rRNA gene, respectively. Details of primer sets and PCR cycling conditions are outlined in Table 1.

**Table 1:** **PCR primer sets for amplification of SSU rRNA region of *Blastocystis *from dog faeces**

**Primer**	**Primer name and sequence (5′ to 3′)**	**Product size**
Single step PCR conditions as per Scicluna *et al*. [14]	BhRDr -GAGCTTTTTAACTGCAACAACG	600 bp
	RD5 –ATCTGGTTGATCCTGCCAGT	
Nested PCR–primary set, conditions as per Clark [13]	RD3-GGGATCCTGATCCTTCCGCAGGTTCACCTAC	1800 bp
	RD5 -GGAAGCTTATCTGGTTGATCCTGCCAGTA	
Nested PCR – secondary set, conditions as per Bohm-Gloning [15], using 1 ul of PCR product from primary step.	Forward -GGAGGTAGTGACAATAAATC	1100 bp
	Reverse - CGTTCATGATGAACAATTAC	

#### Phylogenetic analysis

PCR products were purified using the PureLink Genomic DNA Mini Kit (Life Technologies Corporation, New York, USA) according to the manufacturer’s protocol. Unidirectional DNA sequencing was carried out using the respective reverse primers with an Applied Biosystems 3130/3130xl Genetic Analyzer. DNA sequences were analysed using Finch TV v 1.4.0 (Geospiza Inc., Seattle, WA, USA) and compared with previously published sequences from GenBank (National Center for Biotechnology Information) using Basic Local Alignment Search Tool (BLAST) 2.2.9 [16]. The sequences were aligned with previously published sequences of the SSU rRNA gene of the various *Blastocystis* STs sourced from GenBank using BioEdit v 7.1.3.0 software (Ibis Biosciences, Carlsbad, CA, USA). Neighbour joining analysis and construction of a tree was carried out using Mega 4.1 software (The Biodesign Institute, Tempe, AZ, USA). *Proteromonas lacerate* (U37108) was used as an out-group.

#### Statistical analysis

Prevalence and their 95% confidence intervals for each group of dogs were calculated using EpiTools epidemiological calculators [17].

## Results and discussion

Summary of PCR results are shown in Tables 2 and 3. Of the 80 Indian dogs tested, 19 (24%; 95% CI, 14.4, 33.1) were positive for *Blastocystis* with a predominance of ST1 and ST6. The *Blastocystis* prevalence in the cities of Sikkim, Mumbai and Delhi were 24% (6/25), 14% (4/28) and 33% (9/27) respectively, with dogs in all cities harbouring ST 1, 4, 5 or 6. Of the 80 Brisbane dogs, 2 (2.5%, 95% CI, 0, 5.9) samples were positive, both were from pound dogs and were ST1. Of the 80 Cambodian dogs, 1 (1.3%, 95% CI, 0, 3.7) was positive for ST2. The Brisbane prevalence results of our study are in contrast to those of Duda *et al.*[11] who found, using light microscopy only, that 70.8% of pound dogs from Brisbane harbored *Blastocystis,* a much higher prevalence than the 2.5% described here. This discrepancy could be attributed to the level of care and hygiene of the pound dogs in 1998 compared to current standards, while the source of acquisition and duration of stay within the pound or other confounding factors could have influenced *Blastocystis* prevalence. On the other hand, *Blastocystis* is a morphologically pleomorphic organism and the extensive variation in the appearance of the recognized forms of *Blastocystis* may lead to operator misinterpretations [2,18,19] and in this case, potentially, a high rate of false positives.

**Table 2:** PCR results of dog faecal samples

	**Positive**	***Blastocystis *STs**	**Negative**
**Indian street dogs (80)** (preserved in ethanol)	**19 (24%)**	ST1 – 9	61 (76%)
		ST6 – 7	
		ST4 – 2	
		ST5 – 1	
**Brisbane dogs (80)** (fresh samples)	**2 (2.5%, pound dogs)**	ST1 - 2	78 (97.5%)
**Cambodian dogs (80)** (preserved in 2.5% K_2_Cr_2_O_7_)	**1 (1.3%)**	ST2 – 1	79 (98.7%)

**Table 3:** PCR results of dog faecal samples based on individual primer sets

**PCR**	**Indian street dogs**	**Brisbane dogs**	**Cambodian dogs**
**Single step PCR**	ST1 – 8	ST1 - 2	ST2 – 1
	ST4 – 2		
	ST 5 – 1		
	ST6 – 2		
**Nested PCR**	ST1 – 1	All negative	ST2 – 1
	ST6 – 5		(same sample as above)

*Blastocystis* prevalence can vary between countries and subpopulations, however, the general trend in previous prevalence studies is that developing countries have a higher prevalence than that of developed countries [2]. Prevalence can range from as low as 3.3% in human samples from hospitals in Singapore [20] to 49% in samples from a Nigerian clinic and 70% in children in three counties in Liberia as reported in a ST distribution study by Alfellani *et al.*[8]. Both studies used PCR for diagnosis. In humans, these differences can be attributed to poorer sanitation, higher risk of food and water contamination and also greater exposure to animals and their excrements in developing countries as compared to the developed countries [2,4,21]. Our study, however, did not clearly discriminate between the prevalence of *Blastocystis* between dogs living in developing and developed communities. Mumbai, Delhi and Sikkim have been described as densely populated cities in India with inadequate toileting facilities and sewage systems leaving large sectors of these cities with poor sanitation and hygiene especially in the slum areas [22,23]. As a result, it is estimated that greater than 50% of resource-poor communities within urban areas practice open defecation [23,24]. This may explain the discrepancy between the prevalence of *Blastocystis* in the Indian stray dogs as compared to the Brisbane dogs, however, it does not account for the negligible prevalence of *Blastocystis* in semi-domesticated community dogs in rural Cambodia.

The Indian stray dogs in this study harbored ST 1, 4, 5 and 6 while the Brisbane and Cambodian dogs harbored only ST1 and ST2, respectively. Recently, Alfellani *et al*. [8,25] consolidated the results of multiple studies on *Blastocystis* ST distribution in humans and NHPs in several countries. The most common human STs in the UK for example, were ST3 and 4, while in Africa it was ST1 and 3 [8]. In the NHP study, they found that distribution of ST1, 2 and 3 appeared to be independent of geographical association or NHP group, whereas ST 8 was only observed in arboreal NHPs and species native to Asia or South America [25]. Collectively the results show variable geographical distribution of *Blastocystis* STs in both humans and NHPs between and within subpopulations of different countries. This hypothesis of geographical variation of STs might, at least in part, explain the results in this study, where we found different STs in dogs in different geographical regions except for ST1 which was found in both Indian and Brisbane dogs.

The general low prevalence of *Blastocystis* and the diversity of STs found in dogs in three different geographical regions/ settings suggest that dogs may be transiently and opportunistically infected by whichever *Blastocystis* ST is present in their environment, be it from a human or non-human source. Therefore, dogs are unlikely to act as either natural hosts or primary zoonotic reservoirs for *Blastocystis* but are capable of shedding potentially zoonotic STs and may therefore act as secondary zoonotic reservoirs for infection. The *Blastocystis* STs observed in dogs in this study include STs 1, 2, 4, 5 and 6. ST1 is one of the most common human STs, ST2 is common in UK, Brazil and Central Asia, while ST4 is very common in humans in the UK but rare in other countries [8], though rodents have been shown to be the main animal reservoir of ST4 [1]. ST5 is rare in humans but otherwise commonly reported in ungulates (e.g. pigs, cattle), whereas ST6 is uncommon in humans but otherwise reported mainly in avian species [1,9,26]. Keeping this in mind and the fact that coprophagia is common practice in dogs, the greater prevalence and diversity of STs found in the Indian stray dogs could be attributed to higher population density and greater exposure in their environment to fecal material from human and non-human hosts (cattle, pigs, avians, NHPs), from which they could have either mechanically passaged or acquired opportunistic infections with various *Blastocystis* STs. Ideally, studies to ascertain the relative prevalence of *Blastocystis* STs in humans and animals residing in these three geographical regions especially that of the urban centres in India would provide a clearer epidemiological picture of transmission pathways. Additionally, given that extensive genetic diversity exists within *Blastocystis* STs, ideally future molecular characterisation and comparison of dog, human and other mammalian *Blastocystis* STs using multilocus sequence typing (MLST) performed within communities endemic for *Blastocystis* in dogs and humans will shed further light on their role as natural hosts for infection [27].

## Conclusions

Our results show that a diverse range of *Blastocystis* STs (ST1, 2, 4 ,5 and 6) can be found in dogs and it is likely that there is geographical variation of STs in dogs as has been shown in humans and NHPs [8,25]. Secondly, considering the low prevalence of *Blastocystis* in dogs with no indication of dog-specific/predominant ST, they are unlikely to be natural hosts of *Blastocystis* and are potentially opportunistically infected via coprophagia of other hosts faeces or contaminated drinking water*.* Larger scale *Blastocystis* epidemiological studies on humans and dogs from the same geographical areas with SSU rDNA (18S) allele analysis or MLST would be required to confirm this hypothesis.

## Competing interests

The authors declare that they have no competing interests.

## Authors’ contributions

Conceived and designed the experiments: WW, LC, HBO, HO, RT. Sample collection: WW, TI, RT. Performed the experiments: WW, LC, TI. Analysed the data: WW, LC, HBO, TI, HO, RT. Wrote the manuscript: WW, LC, HBO, HO, RT. All authors read and approved the final manuscript.
